# Patient Engagement and Coaching for Health: The PEACH study – a cluster randomised controlled trial using the telephone to coach people with type 2 diabetes to engage with their GPs to improve diabetes care: a study protocol

**DOI:** 10.1186/1471-2296-8-20

**Published:** 2007-04-11

**Authors:** Doris Young, John Furler, Margarite Vale, Christine Walker, Leonie Segal, Patricia Dunning, James Best, Irene Blackberry, Ralph Audehm, Nabil Sulaiman, James Dunbar, Patty Chondros

**Affiliations:** 1 Department of General Practice, The University of Melbourne, Melbourne, Australia; 2 Department of Medicine, The University of Melbourne, Melbourne, Australia; 3 Chronic Illness Alliance, Melbourne, Australia; 4 Division of Health Sciences, University of South Australia, Adelaide, Australia; 5 School of Nursing, Deakin University, Geelong, Australia; 6 Diabetes Australia Victoria, Melbourne, Australia; 7 College of Medicine, University of Sharjah, Sharjah, United Arab Emirates; 8 Greater Green Triangle University Department of Rural Health, Flinders University and Deakin University, Warnambool, Australia

## Abstract

**Background:**

The PEACH study is based on an innovative 'telephone coaching' program that has been used effectively in a post cardiac event trial. This intervention will be tested in a General Practice setting in a pragmatic trial using existing Practice Nurses (PN) as coaches for people with type 2 diabetes (T2D). Actual clinical care often fails to achieve standards, that are based on evidence that self-management interventions (educational and psychological) and intensive pharmacotherapy improve diabetes control. Telephone coaching in our study focuses on both. This paper describes our study protocol, which aims to test whether goal focused telephone coaching in T2D can improve diabetes control and reduce the treatment gap between guideline based standards and actual clinical practice.

**Methods/design:**

In a cluster randomised controlled trial, general practices employing Practice Nurses (PNs) are randomly allocated to an intervention or control group. We aim to recruit 546 patients with poorly controlled T2D (HbA1c >7.5%) from 42 General Practices that employ PNs in Melbourne, Australia. PNs from General Practices allocated to the intervention group will be trained in diabetes telephone coaching focusing on biochemical targets addressing both patient self-management and engaging patients to work with their General Practitioners (GPs) to intensify pharmacological treatment according to the study clinical protocol. Patients of intervention group practices will receive 8 telephone coaching sessions and one face-to-face coaching session from existing PNs over 18 months plus usual care and outcomes will be compared to the control group, who will only receive only usual care from their GPs. The primary outcome is HbA1c levels and secondary outcomes include cardiovascular disease risk factors, behavioral risk factors and process of care measures.

**Discussion:**

Understanding how to achieve comprehensive treatment of T2D in a General Practice setting is the focus of the PEACH study. This study explores the potential role for PNs to help reduce the treatment and outcomes gap in people with T2D by using telephone coaching. The intervention, if found to be effective, has potential to be sustained and embedded within real world General Practice.

## Background

### The treatment gap and health policy in diabetes

Diabetes has reached epidemic proportions in most countries [1] including Australia [2]. The incidence of diabetes is expected to double worldwide in the next twenty years. The economic costs of diabetes are huge and are doubled by the onset of complications resulting from poor disease control. Diabetes is a complex disorder where multifactorial interventions have been proven to improve disease control and health outcomes. Several major randomised controlled trials (RCTs) have shown reduction of complications with more intensive pharmacotherapy for hypertension, dyslipidaemia and hyperglycaemia in type 2 diabetes (T2D)[3]. A recent study compared intensive hospital-based multi-modality treatment for T2D and associated cardiovascular risk factors with usual treatment in primary care and found a 50% reduction in cardiovascular disease (CVD), nephropathy, retinopathy and neuropathy in the intensively treated group [4]. A range of self-management interventions focusing on patient education and empowerment have also been shown to be effective [5,6].

In Australia, the National Service Improvement Framework (NSIF) for Diabetes states that high quality clinical care and support are critical to improving diabetes outcomes [7]. The framework identifies the characteristics of optimal care as being based on evidence and guidelines, as well as being patient centred, structured to include goal-setting and involving PNs in delivering care. In Australia and the UK, people with T2D receive the majority of their care in General Practice. Yet GPs face barriers to providing structured multidisciplinary care for chronic illnesses such as diabetes. These barriers include the complexity and time consuming nature of chronic disease care as well as a lack of clarity about legitimate goals and outcome measures [8]. GPs' knowledge, skills, attitudes and beliefs may also significantly alter their approach to management [9,10]. In addition, people with diabetes vary in their knowledge and skills, sense of engagement, expectations of treatment, and consequent behaviour. As a result there is often a significant treatment and outcomes gap between clinical diabetes care guidelines and actual clinical practice in General Practice [11,12]. This gap is most marked in socio-economically disadvantaged communities [13-15]. The potential role for PNs to help reduce this treatment and outcomes gap through enhancing patient self-management as well as intensifying therapy, needs further study.

### Educational and psychological interventions

A meta-analysis of 31 RCTs of patient-centred education and self-management interventions in diabetes reported a mean 0.75% absolute reduction in HbA1c [5]. Ismail et al. [6] suggested educational interventions focusing on knowledge and information need to be distinguished from psychological interventions that address individuals' cognitive and emotional functioning and that the latter alone can reduce HbA1c by 1%. Many of the psychological interventions that Ismail et al. reviewed employed Rollnick & Miller's [16] motivational interviewing techniques based on the Transformational Model of Change or counseling strategies focused on Bandura's [17] theory of self-efficacy. Nevertheless, how such interventions have an effect remains unclear [18] although some experts suggest goal setting based on clinical information may be critical [19,20]

### The Coaching patients On Achieving Cardiovascular Health (COACH) intervention

The COACH program uses structured telephone coaching of patients. It is a pragmatic intervention that primes the patient to self-manage by adhering to medication and make relevant behaviour changes. It also, importantly, encourages patients to take greater initiative in the therapeutic alliance, which enables treatment to be appropriately intensified according to clinical practice guidelines in order to achieve treatment goals. The COACH model includes a structured training course for coaches and a series of scheduled and structured telephone sessions with patients addressing lifestyle issues, medication adherence and dosing, how to monitor their disease and how to consult with their GP and allied health services. The COACH intervention has been shown to effectively reduce CVD risk factors in patients with established CVD in a hospital setting using trained, dedicated coaches [21,22]. It is an appropriate model to enhance intensive diabetes treatment because many of the treatment goals are the same. However, it has not been applied or tested in a community setting, nor with PNs acting as coaches. We adapted the COACH intervention for T2D based on information derived from a series of focus groups with people with T2D undertaken to establish whether telephone coaching by PNs would be an acceptable way of managing diabetes for our target sampling population in General Practice. This qualitative phase of the study, which will be reported elsewhere, included a trial run of the PEACH study questionnaires. The PEACH study aims to test whether telephone coaching can reduce the guideline-treatment gap in people with T2D in a way that meets the characteristics of optimum care set out in the NSIF [7].

### Objectives of the study

Our primary objective is to test the effectiveness of the COACH program in improving glycaemic control at 12 and 18 months in patients with poorly controlled T2D (HbA1C >7.5%) compared with usual care only. Our secondary objective is to assess the impact of the intervention on other CVD risk factors (total cholesterol, High Density Lipoprotein (HDL) cholesterol, blood pressure, Body Mass Index (BMI)), behavioural risk factors (exercise, smoking and diet) and process of care measures (achievement of guideline based standards for monitoring, and intensification of therapy). We also plan to study the role of a range of potential explanatory variables, including socioeconomic disadvantage, in predicting outcomes.

## Methods/design

The study is a cluster-randomised, open controlled, intervention trial where General Practices in Melbourne with existing PNs are allocated to one of two groups:

• Intervention group where PNs use the COACH Program to encourage patients with T2D to undertake intensive disease self-management in combination with usual GP care.

• Control group where patients with T2D receive usual GP care only.

### Identification and recruitment of practices and patients

**Figure 1:**
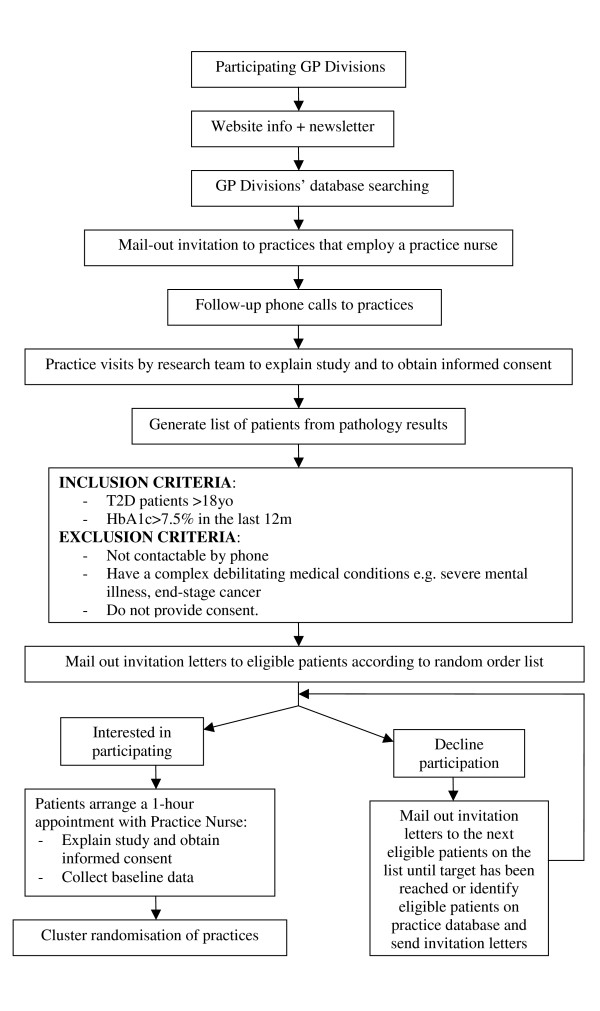
Flowchart of PEACH study recruitment process.

Figure 1 shows the recruitment process of practices and patients into the PEACH study.

#### Practices

GPs are approached using the membership lists of Divisions of General Practice (Divisions of General Practice are geographic organisations of GPs established in 1992 by the Government. They are local organisations that unite GPs and increase their capacity to work co-operatively with each other and other health providers[23]). The study team approached Divisions of General Practice in areas of relatively high socioeconomic disadvantage through metropolitan Melbourne and in 2 large nearby regional centres.

All GPs on the membership list of participating Divisions and who are from practices that employ PNs receive an invitation from the Division with a faxback response sheet. Interested GPs fax back to the research team their intention to participate. Non-responders are reminded by the Division staff with a telephone call one week later. The research team visits practices who express their intention to participate, explain the PEACH study in great detail and provides them with a full information pack describing the aims, methods and expected outcomes of the study, as well as consent form. Consent from all participating GPs and PNs is obtained prior to inclusion in the study. Not all GPs from the practice need to consent to participate for the practice to be included in the study. An information night is held to brief GPs and PNs about the PEACH study implementation.

#### Patients

Participating GPs obtain a list of all diabetic patients with HbA1c ordered by them and measured in the previous 12 months with a value >7.5%, from the local pathology provider. Once a list of eligible patients (see inclusion and exclusion criteria below) have been identified, a random sample of a maximum of 40 patients with HbA1c>7.5% for each practice is sent a letter inviting them to participate in the study, along with a pamphlet about the study, a plain language statement, an expression of interest form, a brief personal survey collecting demographic information and details of duration, treatment and complications of diabetes. The letter is sent using the practice letter head and is signed by the patient's GP and the pack includes a postage paid return stamped envelope addressed to the research team. A member of the research team provides support to the practice in the identification and recruitment of eligible patients. Patients respond by returning the expression of interest form to the research team. All patients, including those who decline, are asked to return the brief personal survey with their response to the letter, allowing a comparison of the participant and non-participant groups. All patients who indicate interest in participating have their details forwarded to their practice and the Practice Nurse contacts the patient to arrange a face-to-face interview at which the study is fully explained and consent is obtained. If patients consent to be included, baseline assessment is undertaken at that face-to-face interview. Patients who have not responded receive a reminder call from the Practice Nurse. If the practice has more than 40 eligible patients, further rounds of randomised mail outs and reminder calls continue until the required number of patients per practice is recruited. A further recruitment strategy of searching practice database to identify eligible patients who had their pathology tests done elsewhere is employed to minimise selection bias and reach recruitment targets of patients with diabetes.

### Inclusion and exclusion criteria

Practices with a practice nurse are eligible to participate, and must achieve a minimum recruitment of five patients to the study to continue. Patients with T2D are eligible to participate if they are >18 years, their most recent HbA1c is greater than 7.5% and they are receiving care from a General Practice that employs a Practice Nurse and is located in one of the participating Divisions of General Practice. Patients are excluded if they are not contactable by telephone, have a complex debilitating coexisting medical condition (e.g. mental illness, end-stage cancer), or do not provide signed consent. Patients from non-English speaking backgrounds are eligible and are able to utilise a free telephone interpreting service to find out more information about the study and throughout the data collection and intervention phases.

### Baseline assessment

Baseline assessment is conducted by the Practice Nurse and takes approximately one hour to complete. Patient written consent is obtained prior to undertaking baseline assessment. Baseline assessment includes data collected by the Practice Nurse at interview (patient knowledge of appropriate testing and goals for risk factors, smoking status, current exercise levels and a dietary history using a validated food frequency questionnaire [24]), from the patient file (treatment, test frequency and most recent pathology results), and patient self report data (diabetes self efficacy scale [25], the diabetes support scale[26], a quality of life scale [27] and a measure of depression [28]). Patients are instructed to arrange baseline testing for HbA1c, total and HDL cholesterol, and renal function at their usual local pathology laboratory. Follow up tests will all be done at the patients' usual pathology laboratory. All participating laboratories use HbA1c assay methods that are DCCT aligned [29] and participate in a regional quality assurance program for HbA1c assays.

### Outcome assessment

Data collected at baseline, including primary and secondary outcome measures, will be collected again at 12 and 18 months post-intervention for both the intervention and control groups. Study team nurses blinded to the group allocation of participants will collect 12 month and 18 month follow up data by face-to-face interview with the patient. We will also access administrative data sets in relation to health service use and cost.

### Randomisation

**Figure 2:**
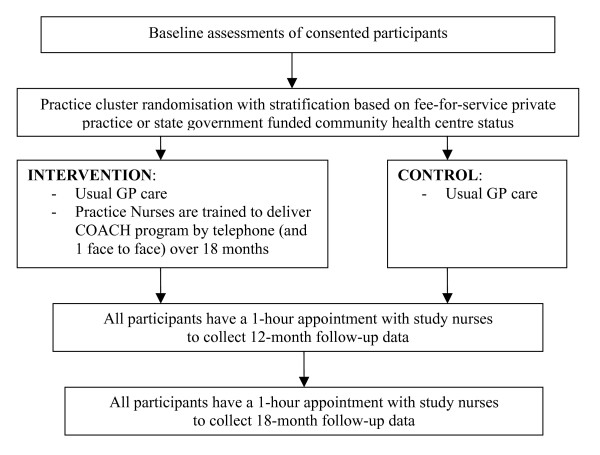
Flowchart of PEACH study intervention process.

General Practices are randomised to either the intervention or control group after all baseline assessments are completed (figure 2). Block randomisation with random block sizes, stratified according to the organisational and financial arrangements of the general practices (fee-for-service private practice or state government funded community health centre status) and whether they are participating in the National Primary Care Collaboratives Program (A national pilot quality improvement program aimed at improving quality of chronic disease care and currently involving approximately 7% of general practices) is used to allocate practices to study groups. The allocation sequence is computer generated by the statistician from the research team, blinded to the identity of the general practices. Following randomisation, practices are informed by a letter from the Chief Investigator of whether their practice will be in the intervention or control group.

### The intervention

Practice Nurses in practices randomised to intervention group undertake a two-day training program in telephone coaching for diabetes using the adapted COACH manual. The telephone coach intervention addresses lifestyle and pharmacological management of hyperglycaemia, and specifically focuses on achieving HbA1c of less than 7% as the goal of treatment. Goals of treatment for the secondary outcomes are also given in table 1. The treatment algorithm given in table 1 is adapted from the Steno-2 Study [30] and is virtually the same as previously used in management of cardiovascular risk factors in patients with coronary heart disease [21,22].

**Table 1:** Treatment algorithm for PEACH study participants adapted from the Steno-2 Study [30].

**Risk Factor**	**Treatment Goal**
HbA1c	<7%
Total cholesterol	<4.0 mmol/L
Blood pressure	<130 mmHg for systolic and <80 mmHg for diastolic
Physical activity	Light to moderate activity for 30 minutes or more on most or all days a week
Smoking	Smoking cessation
Saturated fat	<8% of total daily energy intake
Aspirin	Low dose of aspirin

The COACH Program includes a letter welcoming the patient to the COACH Program and providing written information about coaching sessions. Patients also receive a one-page chart of risk factor targets and a folder with the name and contact details of their Practice Nurse "coach". This folder is designed to store all future COACH Program correspondence. In addition, the Practice Nurse coach places the same one-page chart of risk factor targets and notification of details of patient enrolment in the patient's file for when they next see their GP. The 'Coaching Program' involves five coaching telephone calls at six weekly intervals in the first 6 months, two coaching telephone calls at two monthly intervals between 6–12 months, a face to face coaching session at 12 months and one coaching call at 15 months. Intervention group patients continue to receive usual General Practice care from their GP which can include referral to diabetes educators, dietitians and diabetes specialists that form part of standard diabetes care for patients of that practice.

Patients are coached according to the COACH Model, a process of continuous quality improvement which involves coaching the patient to go to their doctor and obtain measurement of their risk factors and to be informed of the results of these measurements, education regarding risk factor targets, negotiation of a plan of action to achieve the target that includes focus on both medication and lifestyle and subsequent monitoring of progress by the patients toward the achievement of the target level. This quality improvement cycle is a key feature of the COACH Program – each coaching session is used as the foundation for the next coaching session. There is no pre-set time frame for coaching sessions. The length of the calls is determined by the length of time the "coach" needs to establish a plan of action with the patient to be achieved by the next coaching session. Patients are able to contact their Practice Nurse coach between coaching sessions, for questions and further information as required. A specific software package designed for the COACH program is used in conjunction with the telephone coaching sessions to generate written reports for the patient summarising each verbal coaching session, and to inform the "coach" for the next session. Reports are posted to the patients and a copy is placed in each patient's file. Practice Nurses are paid for their training time and for their coaching time.

### Quality assurance

The research team provides on-site assistance to the PN in their first two baseline interviews and questionnaire administration to ensure quality of data collection. All Practice Nurses who deliver the Coach intervention attend the two-day Coach training. In addition, the "Head Coach" (MV) monitors the first telephone coaching and random subsequent telephone coaching. A checklist and regular constructive feedback are provided to all Practice Nurses. A sub-sample of the Practice Nurses, GPs and patients involved in the study will be asked to participate in a semi-structured interview at the conclusion of the study to understand factors influencing variation in implementation of the intervention.

### Sample size calculations

The unit of randomisation in this RCT is the general practice. A total of 546 patients (13 patients per practice) from 42 general practices will be required (273 in each arm) to show an absolute 0.5% or greater reduction in mean HbA1c in the intervention arm compared with usual GP care group. The sample size allows for a design effect of 1.4 due to the increased variance of recruiting patients within practices and an attrition rate of 20% over 18 months and overall attrition rate of 30% over 3 years in anticipation of an extension of the study for another 18 months pending funding. Calculations are based on a two sample t-test for means, with a standard deviation of 1.44 (this is based on results from a previous diabetes project in General Practice in the region involving 1,088 diabetes patients), 80% power and significance level at 5% for a two sided test. The design effect is based on a conservative estimate of the intra-cluster correlation (ICC) for HbA1c of 0.05.

### Data collection, monitoring and analysis

Descriptive statistics will be used to summarise general practice, practice nurse and patient factors for the two study groups and to check for any imbalance in baseline variables between control and intervention group. GP practice will be set as the primary sampling unit. Outcomes between study groups will be compared to assess the effect of the intervention. Primary and secondary outcomes will be analysed using marginal models using Generalised Estimating Equations with information sandwich (robust) standard errors to allow for the effect of clustering of patients within general practices. Analysis will be 'intention to treat' and where appropriate regression analysis will be adjusted for baseline outcome measure and any baseline variables that are imbalanced between the two study groups. Sensitivity analysis will examine the effect of loss to follow-up on the intervention effect. Exploratory analyses are also planned to examine the effect of explanatory factors (e.g. socio-demographic factors such as education level or employment status of patients) on the outcome.

### Trial organization and management

The study has received ethics approval from the Human Research Ethics Committee of the University of Melbourne. No significant risks to participants are anticipated. As the study is unblinded and low risk a data monitoring committee is unnecessary.

A study reference group consisting of representatives of the three main Divisions of General Practice involved, as well as Diabetes Australia (a national not-for profit organisation offering assistance to people with diabetes, to health professionals and providing input into national policies) and the Australian Practice Nurses Association has been formed and meets quarterly for the duration of the study to provide important contextual advice. Eight T2D patients who have participated in the qualitative phase formed a consumer reference group and will meet biannually to provide additional consumer input to the study. The chief investigators meet monthly to oversee implementation of the study.

## Discussion

One of the strengths of this study is that it is evaluating an intervention that links a combined educational and psychological intervention focusing on both self management and a strengthening of the therapeutic alliance in intensifying treatment. This combination of strategies and the focus of the telephone coaching maximises the potential impact of our intervention based on preceding reviews of non-pharmacological interventions [5,6] and the demonstrated efficacy of intensive medical therapy[31]. The principles behind the modified COACH program have been tested for face validity as an acceptable mode of diabetes care in focus groups in the early phases of our study. Telephone coaching was seen as an acceptable adjunct to usual practice based care. A further strength of our study is that it specifically sets out to recruit patients from areas of socioeconomic disadvantage and non-English speaking backgrounds. These groups are often not well represented in intervention studies for a range of reasons, making the findings less generalisable to these groups. Our study will potentially provide evidence of effectiveness of an intervention not only for reducing the treatment and outcomes gap in diabetes care, but also for improving equity of health outcomes.

The setting in general practice and the use of existing practice nurses in a cluster randomised design ensures rigour of evaluation design but also relevance and generalisability of the findings to the general practice setting. The present study also provides the opportunity to study an intervention over a longer period of time than is usual in trials of this nature. Reviews have found that the effect of many chronic disease interventions is not sustained when contact for the intervention finished[5,32]. In the previous study of coaching for CVD in hospital patients cholesterol lowering in the intervention group was also not maintained when coaching was completed (unpublished data M Vale, J Best). Critical to this notion of sustainability is the setting and embedded nature of our study. Basing the study in General Practices and using existing Practice Nurses as coaches offers the opportunity to develop evidence on the organisation of work and roles for the rapidly expanding Practice Nurse workforce. Our study will test the potential for integrating the program into the General Practice setting and to maximise cost-effectiveness and sustainability.

## Competing interests

The author(s) declare that they have no competing interests.

## Authors' contributions

DY, JB and MV conceptualised the research. DY, JB, JF, MV, NS, CW, L S, PC, JD, RA, and TD conceived the development of study design. DY, IB and JF oversaw the running of the trial and drafted the protocol. MV, JB, TD and RA adapted the COACH intervention for the PEACH study.

All authors read and approved the final manuscript.

## Pre-publication history

The pre-publication history for this paper can be accessed here:


